# The prevalence and nutritional status of adolescent Saudi girls with disordered eating

**DOI:** 10.1017/jns.2022.71

**Published:** 2022-08-26

**Authors:** Ahlam Badreldin El Shikieri

**Affiliations:** College of Applied Medical Sciences, Clinical Nutrition Department, Taibah University, P.O.Box 4583, Al Madinah Al Munawarah 41412, Saudi Arabia

**Keywords:** Adolescent girls, Eating disorders, Nutritional status, Prevalence, Saudi Arabia, AN, anorexia nervosa, ANOVA, analysis of variance, BED, binge eating disorders, BN, bulimia nervosa, CI, confidence interval, DSM-5, Diagnostic Statistical Manual of Mental Disorders, Fifth Edition, EAT-26, Eating Attitudes Test-26, ED, eating disorders, non-ED, non-eating disordered, OSFED, other specified feeding or ED, SPSS, Statistical package for social sciences software, UFED, unspecified feeding or ED, WHO, World Health Organization

## Abstract

This study on adolescents was intended to assess the prevalence of disordered eating attitudes and the nutritional status of adolescent girls in Saudi Arabia. Disordered eating attitudes and behaviour were assessed using the EAT-26. The type of eating disorder (ED) was determined using Diagnostic statistical manual of mental disorders, fifth edition. The nutritional status of the adolescent girls was determined by measuring their weight and height twice using standard protocols. The BMI-for-age and height-for-age were defined using WHO growth charts. Comparisons between adolescent girls with and without EDs were conducted using SPSS version 26. Eating disorders (EDs) were prevalent among 10⋅2 % of these girls. Other specified feeding or EDs were the most prevalent ED (7⋅6 %), followed by unspecified feeding or eating disorder (2⋅4 %). Anorexia nervosa was common among 0⋅3 % of the girls. The eating disordered adolescents were either overweight (7⋅7 %), obese (10⋅3 %), stunted (7⋅7 %) or severely stunted (2⋅6 %). ANOVA revealed that the BMI-for-age was influenced by age (*P* = 0⋅028), the type of ED (*P* = 0⋅019) and the EAT-26 (*P* < 0⋅0001). Pearson's correlation showed that the EAT-26 score increased significantly with the BMI (*r* 0⋅22, *P* = 0⋅0001), height (*r* 0⋅12, *P* = 0⋅019) and weight (*r* 0⋅22, *P* = 0⋅0001). The early detection of EDs among adolescents is highly recommended to reduce the risk associated with future impaired health status. Nutrition professionals must target adolescents, teachers and parents and provide nutritional education about the early signs and symptoms of ED and the benefits of following a healthy dietary pattern.

## Introduction

The World Health Organization (WHO) defines adolescents as children aged between 10 and 19 years old^(1)^. Their nutritional status dictates their development and health outcomes, including their mental health. Eating disorders (EDs) are psychiatric illnesses described as habitual disturbances in eating and weight-control attitudes with diagnostic criteria based on psychological, behavioural and physiological characteristics unrelated to medical or psychiatric factors^(2)^. They are the most common psychiatric problems among adolescents.

Various types of EDs have been reported among adolescents. Anorexia nervosa (AN) is characterised by an intense fear of fatness or seeking low body weight and the disturbance of body image^(3)^. The risk factors among this age group include a family history of AN and bulimia nervosa (BN), dissatisfaction regarding parents’ weight and shape, perfectionism and significant concern about feeling fat^(4)^. Unspecified feeding or eating disorder (UFED) is present when behaviours cause clinical distress or impairment of functioning. However, the symptoms do not meet the full criteria of any of the feeding or ED criteria, such as AN, BN and binge eating disorders (BED)^(5)^.

Moreover, individuals with the other specified feeding or eating disorder (OSFED) type have either subthreshold symptoms of anorexia or bulimia, mixed features of both disorders or highly atypical eating behaviours. The latter occurs when all the criteria for AN are met, without significant weight loss; the weight might be within or above the normal range^(5,6)^.

Furthermore, there are variations in the prevalence and types of ED among adolescents worldwide, including the Arab world^(7)^. For instance, the prevalence of EDs among adolescent girls in Arar and Jeddah was 25⋅5 and 33 %, respectively^(8,9)^. Additionally, studies revealed that the ED risk is twice as high among girls than boys in Jordan, Libya, Palestine and Syria. Adolescent boys in Kuwait had a higher prevalence of disordered eating attitudes (47⋅3 %) than their counterparts (42⋅8 %)^(7)^. Among Chinese adolescents, the prevalence of the various types of ED was 1⋅05 % for AN, 2⋅98 % for BN and 3⋅58 % for BED. The prevalence in Africa was lower: AN was <0⋅01 %, BN was 0⋅87 % and BED was 4⋅45 %^(10)^. Notably, Europe had the highest percentages of EDs, ranging from 1 to 4 % for AN, 1–2 % for BN and 1–4 % for BED^(10)^.

EDs have adverse health effects. Among adolescents, EDs can lead to psychological and permanent physical consequences^(11)^. Earlier studies have indicated that EDs are associated with an increased risk of overweight/obesity and cardiovascular diseases^(12)^. A study in 2017 that assessed the risk of obesity/overweight among adolescents with EDs based on their BMI-for-age revealed high rates of overweight and obesity^(7)^. In the AFINOS study, among 195 adolescents (97 girls) aged 13–18 years old, 24⋅7 % were at risk of an ED, and 21⋅6 % suffered from being overweight^(13)^.

A literature search revealed a limited number of published studies focusing on malnutrition among adolescents with EDs. In our study, malnutrition refers to two conditions: excessive nutrition (overweight and obesity) and its deficiency (wasting, stunted and underweight). Therefore, the aim of the current study was to (1) determine the prevalence of disordered eating attitudes among adolescent Saudi girls and (2) assess the nutritional status of ED girls. It is hypothesised that EDs are common among Saudi adolescent girls, and they suffer from impaired nutritional status.

## Method

### Study design and participant selection

An epidemiological, cross-sectional, descriptive, community-based comparative study was conducted in Al Madinah Al Munawara, Saudi Arabia, between February and May 2017. Public and private female elementary, intermediate and high schools were targeted to recruit adolescents aged 10–18. Adolescent girls were included irrespective of their socioeconomic status. A list of all girls’ schools was obtained from the Ministry of Education website. The names of the schools were entered into a ballot to select an equal number of public and private schools in the Al Madinah Al Munawarah region. The schools’ headmistresses were then approached with letters issued by the Dean of the College of Applied Medical Sciences at XXX University.

A multistage stratified sampling technique was followed to select the adolescent girls from each school. Girls were randomly selected based on the number of girls aged 10–18 years old in each school, class and sampling fraction. The parents of the chosen girls were sent invitation letters explaining the aim of the study, its duration and the parameters that will be assessed. Their consent was sought before the start of the study. Girls with chronic diseases, such as diabetes mellitus, hypertension, hypo/hyperthyroidism and who were pregnant or lactating were excluded. In these cases, another name from the list of students was selected. A flowchart summarises the recruiting process in Supplementary Figure 1.

### Defining and diagnosing EDs

The Eating Attitude Tests 26 (EAT-26) determined the risk of girls’ disordered eating attitudes and behaviours. It was previously validated in a study conducted in Saudi Arabia^(14)^. It has three sets of questions. In Part A, information about current and ideal body weight was obtained. Part B included three subscales, dieting, bulimia and food preoccupation and oral control, which were questioned with a total of 26 items. Each item (except the 26th item) had six response options ranging from 0 to 3. Lastly, in Part C, four behavioural questions were asked to determine extreme weight-control behaviours and estimate their frequency, e.g., self-induced vomiting over the preceding 6 months. Overall, females who scored ≥20 on the EAT-26 were classified as ‘at-risk’ for disordered eating attitudes and behaviours^(15,16)^.

### Diagnostic statistical manual of mental disorders, fifth edition

Based on the EAT-26 scores, girls diagnosed with EDs completed the Diagnostic statistical manual of mental disorders, fifth edition (DSM-5) to specify the type of ED^(17,18)^. The DSM-5 is a 23-component self-reported questionnaire identifying EDs, namely, AN, BN, BED, OSFED and UFED. The first three are the *typical* EDs, and the others are known as *atypical* forms of ED^(6)^. The questionnaire starts with questions about physical appearance and how shape influences judgement as a person. The scores ranged from zero to six, where zero means not at all (not suffering from this problem), and six means extremely (highly suffering from the problem). Then, questions were asked about eating episodes with a loss of control, feeling during and after overeating, e.g., eating much more and more rapidly. The DSM-5 also included information about the highest weights at the current height.

### Anthropometric measurements

Participant weight and height were measured twice following standard methods. The weights were measured using a digital scale (Beurer GmbH. Söflinger Srt.218, Germany), which measured up to 150 kg. Girls were asked to take off their shoes and any heavy objects from their pockets. Weights were recorded to the nearest 0⋅1 kg. Heights were measured to the nearest 0⋅1 cm by a stadiometer (Seca 213 Portable stadiometer Height-Rod), which measures up to 200 cm. Girls were further asked to take off their shoes and stand with their backs against the board and their eyes perpendicular to the ground. Their BMIs were then calculated using the Quetelet equation:
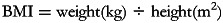


The classifications of body measurements were obtained using WHO growth charts based on *z* scores ^(19)^. For the height classification, height-for-age tables were used. Heights that fell between −1 and 2 standard deviations (sd) were classified as normal; −2 was classified as stunted and −3 was classified as severely stunted.

BMI-for-age tables were used to classify the weights of the adolescents. Values that fell at plus 3 were considered obese, plus 2 as overweight, 1 as possible risk of overweight, minus 1 up to zero as normal, minus 2 as wasted and minus 3 as severely wasted. A point that fell between the z score lines ‘minus 2 and minus 3’ was counted as ‘below minus 2’, whereas a point between ‘plus 2 and 3’ was considered ‘above 2’^(20)^.

Ethical permission to conduct the current study was obtained from the Ethical Committee at the College of Applied Medical Sciences number CLN 201 703. Ethical approval was also obtained from the different schools’ authorities before the beginning of the study. The adolescents’ parents gave their consent before the start of the study, and they were assured that all the information would be maintained as private. They were further informed that they had the right to withdraw from the study without any pressure. Eating disordered adolescents were advised to contact their paediatricians for a detailed check-up.

In the current study, the exposure variable was nutritional status as defined by anthropometric measurements, whereas the various types of EDs were the outcome variables.

### Quality control

Data collectors were trained before the start of the study. Their training included recruiting adolescents, using the EAT-26 and DSM-5, and measuring body weights and heights. The aim of the training was to reduce any data collection errors. The data collectors also ensured that there was no missing information. The data collection tools were pretested on ten students before the start of the study to check the clarity of the questions. In addition, the weighing scales were calibrated daily. When the values were not within acceptable ranges, they were considered outliers and removed from the analysis. For such cases, another measurement was taken either for the anthropometric measurements or by repeating the DSM-5.

### Data analysis

Descriptive and inferential data were analysed using Statistical Package for the Social Sciences software (SPSS, version 26). Pearson's correlation was used to determine the association between the anthropometric measurements and other variables, such as age. An analysis of variance (ANOVA) was used to assess the differences between ED and non-ED individuals for their BMI-for-age and height-for-age. An independent Student's *t*-test determined the differences between the ED and non-ED adolescents regarding their age, EAT-26 scores, and anthropometric measurements. Adolescents with an EAT-26 ≥ 20 would be referred to in the tables as eating disordered (ED), and those with an EAT-26 score of <20 would be referred to as non-eating disordered (non-ED). Logistic Binary Regression was used to predict the risk of having ED using EAT-26 scores as dependent variables, and the independent variables were BMI-for-age and age groups. A *P* value of ≤5 % was set as the significance level.

## Results

Adolescent girls (*n* 396) aged 10–18 years were recruited from different schools in Al Madinah Al Munawarah. Some of these adolescents (*n* 8) were excluded for not fulfilling any of the DSM-5 criteria. Seven were further excluded because their parents refused to allow their daughters to complete their study. Overall, 381 girls were included, with a response rate of 96⋅2 %.

According to the EAT-26 score, some girls (*n* 39, 10⋅2 %) were at risk for disordered eating attitudes and behaviours. As expected, their scores were significantly higher than those of girls with normal eating attitudes and behaviours. The most prevalent type of ED was OSFED, namely, atypical AN (*n* 29, 7⋅6 %), followed by UFED (*n* 9, 2⋅4 %), and only one girl suffered from AN (0⋅3 %), as determined by the DSM-5. None of the girls suffered from BN or BED. Thus, the study's first hypothesis, which stated that EDs are prevalent among adolescent girls, was accepted.

Moreover, girls with EDs were heavier (based on their weight) and taller (based on their height) and had a significantly higher BMI than their counterparts (Table 1). Many ED adolescent girls had a possible risk of being overweight, and a significant number were obese (Table 2). Overall, girls without EDs (7 %) were either wasted or severely wasted, whereas none of those with EDs suffered from these conditions. Additionally, 10 % of the total girls (*n* 381 adolescent girls) suffered from stunted and severely stunted growth (Table 2). Thus, the study's hypothesis, which stated that eating disordered females suffered from over- and undernutrition, was accepted.

**Table 1.** Age, EAT-26^a^ scores and anthropometric measurements among Saudi adolescents (*n* 381)

	ED (*n* 39)	Non-ED (*n* 342)	Total (*n* 381)	*P* value
Age (years)^b^	13⋅6 (2⋅6)	13⋅1 (2⋅7)	13⋅1 (2⋅7)	0⋅20
1^st^ Quartile	11	11	11	
2^nd^ Quartile	13	12	12	
3^rd^ Quartile	16	15	15	
95 % CI	−0⋅35, 1⋅46	−0⋅33, 1⋅44		
EAT-26 score^b^	29 (7⋅5)	11⋅0 (5⋅0)	13⋅0 (7⋅5)	<0⋅0001
1^st^ Quartile	22	7	8	
2^nd^ Quartile	27	11	12	
3^rd^ Quartile	33	15	16	
95 % CI	16⋅1, 19⋅6	15⋅4, 20⋅4		
Weight (kg)^b^	51⋅5 (13⋅4)	42⋅7 (12⋅3)	43⋅6 (12⋅7)	<0⋅0001
1^st^ Quartile	39⋅8	33	33⋅6	
2^nd^ Quartile	54⋅3	42⋅3	42⋅7	
3^rd^ Quartile	62⋅4	50	51⋅4	
95 % CI	4⋅64, 12⋅91	4⋅24, 13⋅31		
Height (m)^b^	1⋅51 (0⋅1)	1⋅46 (0⋅1)	1⋅46 (0⋅1)	0⋅014
1^st^ Quartile	1⋅41	1⋅40	1⋅39	
2^nd^ Quartile	1⋅54	1⋅47	1⋅47	
3^rd^ Quartile	1⋅59	1⋅54	1⋅54	
95 % CI	0⋅01, 0⋅08	0⋅01	0⋅08	
BMI (kg m^−2^)^b^	22⋅4 (3⋅9)	19⋅7 (4⋅3)	20 (4⋅3)	0⋅0001
1^st^ Quartile	19⋅4	16⋅8	17	
2^nd^ Quartile	23⋅0	19⋅0	19⋅2	
3^rd^ Quartile	24⋅8	22⋅0	22⋅4	
95 % CI	1⋅22, 4⋅05	1⋅29, 3⋅98		

a EAT-26: Eating Attitudes Test-26. Those with scores ≥20 suffered from ED.

b Geometric mean and standard deviation; Values are numbers and percentages. The total number of adolescents was 381.

**Table 2.** BMI-for-age and height-for-age classifications among Saudi adolescents (*n* 381)

	ED (*n* 39)	Non-ED (*n* 342)	Total (*n* 381)	*P* value
BMI-for-age classification^a^				0⋅0001
Normal	18 (46⋅0)	216 (63⋅2)	234 (61⋅0)	
Possible risk of overweight	14 (36⋅0)	60 (17⋅5)	74 (19⋅4)	
Overweight	3 (7⋅7)	36 (10⋅5)	39 (10⋅2)	
Obese	4 (10⋅3)	6 (1⋅8)	10 (2⋅6)	
Wasted	−	16 (4⋅7)	16 (4⋅2)	
Severely wasted	−	8 (2⋅3)	8 (2⋅1)	
Height-for-age classification^a^				0⋅75
Normal	35 (89⋅7)	307 (89⋅9)	342 (89⋅9)	
Stunted	3 (7⋅7)	31 (9⋅3)	34 (8⋅9)	
Severely stunted	1 (2⋅6)	4 (1⋅2)	5 (1⋅3)	

a Values are numbers and percentages. The total number of adolescents was 381.

Furthermore, the only anorexic adolescent girl was older and taller than her counterparts (Table 3). Age and height significantly differed among the various ED types, with those with OSFED being older and taller than those with UFED. Although the BMI-for-age tended to be significantly different among the three types of ED, more than half of those with UFED were overweight or obese. Those with OSFED were either at possible risk of being overweight, overweight or obese (Table 3). Stunted growth is also prevalent among those with OSFED.

**Table 3.** Anthropometric measurements as classified by the type of ED among Saudi adolescents (*n* 39)

	AN (*n* 1)	OSFED (*n* 29)	UFED (*n* 9)	*P* value
Age (years)^a^	17 (−)	14⋅1 (2⋅3)	11⋅4 (2⋅2)	0⋅007
Weight (kg)^a^	69⋅0 (−)	52⋅8 (13⋅4)	45⋅3 (11⋅7)	0⋅14
Height (m)^a^	1⋅59 (−)	1⋅54 (0⋅1)	1⋅39 (0⋅1)	0⋅001
BMI (Kgm^−2^)^a^	27⋅3 (−)	22⋅0 (4⋅1)	23⋅0 (3⋅3)	0⋅37
BMI-for-age classification^b^				0⋅08
Normal	−	14 (48⋅3)	4 (44⋅4)	
Possible risk of overweight	1 (−)	13 (44⋅8)	−	
Overweight	−	1 (3⋅4)	2 (22⋅2)	
Obese	−	1 (3⋅4)	3 (33⋅3)	
Height-for-age classification^b^				0⋅72
Normal	1 (−)	26 (89⋅7)	8 (89)	
Stunted	−	3 (10⋅3)	−	
Severely stunted	−	−	1 (11)	

DSM-V, determined types of ED; AN, anorexia nervosa; OSFED =, other specified feeding or ED; UFED, unspecified feeding or ED.

a Geometric mean and standard deviation.

b Values are numbers and percentages. The total number of adolescents was 39.

ANOVA revealed that the BMI-for-age was influenced by age (*P* = 0⋅028), the type of ED (*P* = 0⋅019) and EAT-26 (*P* < 0⋅0001). Pearson's correlation showed that the EAT-26 score increased significantly with the BMI (*r* 0⋅22, *P* = 0⋅0001), height (*r* 0⋅12, *P* = 0⋅019) and weight (*r* 0⋅22, *P* = 0⋅0001). In addition, Logistic Binary Regression revealed that girls who were either at possible risk for overweight (Odd ratio = 1⋅34, 95 % CI 0⋅157, 0⋅723, *P* = 0⋅005) or were obese (Odd ratio = 1⋅38, 95 %CI = 0⋅023, 0⋅382, *P* = 0⋅001) had higher probabilities for developing EDs as defined by their EAT-26 scores.

## Discussion

Adolescents are future productive adults. Their development and healthy growth depend on the quality and amount of food consumed and future health consequences^(21)^. Puberty is a more critical and challenging time for adolescent girls than for boys. One of these challenges is their increased risk for EDs. Possible risk factors include early exposure to family, culture and peer pressure^(22,23)^. Earlier studies suggested a positive association between EDs among adolescents and an increased risk of obesity, cardiovascular diseases and other mental health disorders^(12)^.

The aim of the current study was to assess the prevalence of ED and the nutritional status of adolescent girls with disordered eating. Our findings revealed an ED prevalence of 10 % among girls in Al Madinah Al Munawarah. Contrary to our study findings, the prevalence of EDs among adolescents aged 15–18 in Jeddah city, Saudi Arabia, was higher (33 %)^(8)^. Additionally, higher prevalences were reported among Slovakian (age: 11–15 years; 37⋅2 %)^(24)^ and Spanish (age: 13–18 years; 24⋅7 %)^(13)^ counterparts. However, the prevalence of EDs in our study was higher than among Singaporean (7⋅4 %)^(25)^ and Ethiopian (6 %) adolescents^(26)^.

Moreover, the most prevalent types of EDs in our study were OSFED (7⋅6 %) and UFED (2⋅4 %), with one girl suffering from AN (0⋅3 %). AN was not expected among Saudi adolescents. Previous studies in Saudi Arabia reported that religious and cultural factors forbid people from experiencing self-starvation^(27)^. In the past, AN was considered a Westernised disorder not prevalent among non-Western countries^(28)^. Compared with our study, the prevalence of AN was lower than among Iranian 13- to 18-year-old adolescents [1⋅3 %], whereas OSFED was higher (1⋅7 %)^(29)^. Additionally, the prevalence of OSFED among Jordanian 10- to 16-year-old adolescents was higher than that in our study (31 % *v*. 7⋅6 %, respectively)^(30)^. The possible explanation for this difference could be related to the fact that adolescents focus more on their body weight, as encouraged by various media messages. Interestingly, our study found no correlation between age and EAT-26 scores, as reported previously^(8)^. However, our findings revealed significant differences in age among the various types of ED, with those having OSFED being older.

Furthermore, abnormal eating attitudes are common among adolescents with overweight and/or obesity^(31)^. Childhood obesity is a major risk factor for future chronic diseases, including diabetes mellitus, hypertension, impaired lipid profile and polycystic ovarian syndrome. Our study revealed that eating disordered adolescent girls were heavier based on their BMI-for-age. The differences in BMI-for-age between ED and non-ED adolescents tended to be significant (*P* = 0⋅08). Our findings were similar to findings among Brazilian adolescents^(10)^. The mean BMI for ED adolescents in our study was similar to that of their counterparts in Madrid (21⋅7 kgm^−2^)^(13)^ and Jeddah (21⋅7 kgm^−2^)^(8)^. Additionally, our study showed that EAT-26 scores increased significantly with BMI. Similar findings were reported previously in Jeddah^(8)^, Madrid^(13)^ and Slovakia^(24)^. Being overweight pre- or during adolescence might lead adolescents to adopt disordered eating attitudes to lose excessive weight^(32)^.

Furthermore, malnutrition during puberty is associated with weak bones and permanently stunted height^(33)^. Stunting among adolescents is often underrecognised^(11)^. Stunted growth is more prevalent among eating adolescents suffering from AN, leading to long-term complications^(34)^. Growth spurts among adolescents depend on overall health status and the duration of malnutrition^(33)^. There was only one girl who suffered from AN in our study, and she was not stunted. However, 10 % of the OSFED girls were stunted, and 11 % of the UFED were severely stunted. The low height-for-age among adolescents indicates that these girls were suffering from malnutrition for a long time before their heights were affected. Thus, previous nutritional intake and psychosocial factors might be associated with disordered eating and stunted growth^(35)^. Our study was not consistent with previous studies, revealing that stunted growth is more prevalent among anorexic adolescents. This result could be attributed to the small number of anorexic girls in our study^(29)^.

The current study is considered the first of its kind to focus on both under- and overnutrition among adolescent girls with disordered eating in Al Madinah Al Munawarah, Saudi Arabia. Previous studies focused only on overweight and obesity and their occurrence among ED adolescents. In addition, anthropometric measurements were taken twice, and the validated version of EAT-26 was used. These considerations have reduced the bias associated with data collection and gave high credibility to the findings. Hence, the methods used here were both valid and reliable. However, the main limitation of our study is its cross-sectional design, which will not lead to causal relationships. Furthermore, although the sample was selected randomly, the limited sample size makes it difficult to generalise the findings.

In conclusion, the prevalence of malnutrition and the risk of ED in our study were comparable with previous studies in Western and other non-Western societies. Among adolescent Saudi girls, overweight and obesity are associated with EDs. Some girls also suffered from stunted growth. However, our findings should be taken with caution because they are only valid for girls in Al Madinah Al Munawarah aged 10–18 years old. Therefore, the findings could not be generalised to include other Saudi regions or younger girls. The current study also highlighted the critical role that nutrition professionals should play in the community. Efforts should be made to detect the early signs and symptoms of EDs among adolescent girls. In addition, nutrition professionals should develop educational programs targeting girls, teachers and parents, focusing on the early signs and symptoms of EDs and ways to prevent their development. Following a healthy dietary pattern will also prevent the development of EDs. Hence, it is crucial and necessary to develop educational and public health programs to identify, prevent and treat EDs among adolescents from a broad perspective.

## Supplementary material

For supplementary material accompanying this paper visit https://doi.org/10.1017/jns.2022.71. click here to view supplementary material
