# Supplementary material for: The prevalence and nutritional status of adolescent Saudi girls with disordered eating
Source: J Nutr Sci. 2022 Aug 26;11:e71. doi: 10.1017/jns.2022.71 (PMC9428658; doi:10.1017/jns.2022.71)
Supplement: Supplementary file 1 [file S2048679022000714sup001.docx]

Flow chart for the selection of the girls, “Online Supplementary Material”

**List of public and private elementary, intermediate and high schools in Al Madinah Al Munawarah.**

**Randomly selected 6 private and 6 public schools. The total number of girls aged 10-18 years was 396.**

**All selected schools were contacted formally by a letter addressed by the Dean of the Faculty of Applied Medical Sciences to the Headmistress.**

**In each school, a list of girls aged 10-18 years was obtained from the Head teacher of the class. Girls were then selected randomly using a fraction based on the number of girls in each class (every $n^{th}$ girl).**

***Inclusion criteria***

**Girls aged 10-18 years; Not suffering from chronic conditions such as diabetes mellitus, hypertension, hypo/hyper-thyrodism. In addition, those who were pregnant and lactating, were not included. In total, 15 girls were excluded. Thus, the total number of girls included in the study was 381.**
